# Omental adipose tissue gene expression, gene variants, branched-chain amino acids, and their relationship with metabolic syndrome and insulin resistance in humans

**DOI:** 10.1007/s12263-014-0431-5

**Published:** 2014-09-27

**Authors:** Aurora E. Serralde-Zúñiga, Martha Guevara-Cruz, Armando R. Tovar, Miguel F. Herrera-Hernández, Lilia G. Noriega, Omar Granados, Nimbe Torres

**Affiliations:** 1 Departamento de Fisiología de la Nutrición, Instituto Nacional de Ciencias Médicas y Nutrición Salvador Zubirán, Vasco de Quiroga No. 15 Tlalpan, 14000 México, DF Mexico; 2 Departamento de Cirugía, Instituto Nacional de Ciencias Médicas y Nutrición Salvador Zubirán, México, DF Mexico

**Keywords:** Branched-chain amino acid, Omental, White adipose tissue, Gene expression, Polymorphisms, Metabolic syndrome, Obesity

## Abstract

Obesity is a complex disorder caused by several factors. Thus, the aim of the present study was to assess whether the expression of genes in the omental white adipose tissue (AT) of subjects with insulin resistance (IR) or metabolic syndrome (MetS) is associated with an elevation in serum branched-chain amino acids (BCAAs) and whether this response depends on specific genetic variants. Serum BCAA concentration, the adipocyte area, and gene variants of *PPARγ, ABCA1, FTO, TCF7L2, GFOD2,* *BCAT2*, and *BCKDH* were determined in 115 Mexican subjects. The gene expression in the AT and adipocytes of *BCAT, BCKDH E1α, C/EBPα, PPARγ2, SREBP*-*1, PPARα, UCP1, leptin receptor, leptin, adiponectin*, and *TNFα* was measured in 51 subjects. Subjects with IR showed higher values for the BMI, HOMA-IR, and adipocyte area and higher levels of serum glucose, insulin, leptin, and C-reactive protein, as well as an elevation of the AT gene expression of *SREBP*-*1, leptin*, and *TNFα* and a significant reduction in the expression of *adiponectin, BCAT2*, and *BCKDH E1α*, compared with non-IR subjects. The presence of MetS was associated with higher HOMA-IR as well as higher serum BCAA concentrations. Subjects with the genetic variants for *BCAT2* and *BCKDH E1 α* showed a lower serum BCAA concentration, and those with the *ABCA1* and *FTO* gene variant showed higher levels of insulin and HOMA-IR than non-IR subjects. AT dysfunction is the result of a combination of the presence of some genetic variants, altered AT gene expression, the presence of MetS risk factors, IR, and serum BCAA concentrations.

## Introduction

The incidence of obesity has increased dramatically in recent decades. Omental white adipose tissue (AT) dysfunction is a primary defect in obesity and may link obesity to several health problems, including increased risk for insulin resistance (IR), type 2 diabetes, fatty liver disease, hypertension, dyslipidemia, and some cancers. For obese men (BMI ≥ 30 kg/m^2^), the risk of developing type 2 diabetes is 9-fold higher than for lean men (Weinstein et al. 2004). Adipocyte dysfunction could be caused by different factors, including the presence of specific gene variants, disequilibrium in adipocyte gene regulation, the presence of risk factors for metabolic syndrome (MetS), and the presence of some other metabolites. Previous reports have described an association between branched-chain amino acids (BCAAs) and IR and a strong correlation between serum BCAA and obesity (Newgard et al. 2009; Tai et al. 2010) and have indicated that BCAA concentration is a predictor of the progression of diseases such as diabetes (Shah et al. 2012; Wang et al. 2011).

However, it is not known how the levels of BCAAs increase in subjects with obesity and IR; several possibilities do exist, though. One contributor could be the tissue-specific expression of genes encoding key BCAA catabolism enzymes. The main amino acid-catabolizing enzyme in BCAA catabolism is the mitochondrial branched-chain aminotransferase (*BCAT2*), which catalyzes the transamination of the three BCAAs, leucine, isoleucine, and valine, to the corresponding branched-chain keto acids. A second enzyme, branched-chain keto acid dehydrogenase, catalyzes the formation of the corresponding acyl CoA derivatives from the branched-chain keto acids (Harper et al. 1984). Interestingly, recent findings have demonstrated that AT contributes to the alterations of BCAA metabolism of metabolically compromised individuals compared to subcutaneous adipose tissue indicating the importance of AT in the modulation of circulating BCAA levels (Lackey et al. 2013). The second contributor to the higher serum BCAA during obesity could be the presence of specific gene variants of BCAA enzymes, although this contribution must still be elucidated. Studies have reported that the frequency of the genetic variants *BCKDH* and *BCAT2* is 25 and 12 %, respectively, in the American population (EMBL-EBI 2013). The third contributor could be AT dysfunction. AT plays an important role in BCAA catabolism because this organ has a large capacity for transamination and oxidative decarboxylation of BCAA after skeletal muscle (Brosnan and Brosnan 2006). For this reason, AT could be a key player in obesity-related metabolic dysfunctions. Thus, the aim of the present study was to understand the role of BCAA metabolism in IR and MetS and to provide a global view of the interactions between genetic variants, inflammation, IR, BCAA metabolism, cytokines, and AT gene expression.

## Materials and methods

### Participants

This study was performed at the Department of Physiology of Nutrition of the Instituto Nacional de Ciencias Médicas y Nutrición, Salvador Zubirán (INCMNSZ), Mexico City. We included Mexican Mestizos subjects of both genders, with ages between 18 and 65 years, who underwent an elective laparoscopic surgery. AT of control subjects was obtained from patients that underwent Nissen fundoplication for hiatus hernia, Heller cardiomyotomy type for achalasia, and elective cholecystectomy for chronic cholecystitis. Biopsies of the intra-abdominal omental fat depots were collected in Krebs–Ringer buffer. Mexican Mestizos were considered only those individuals who had been born in Mexico for three generations. These individuals are descendant of the original autochthonous inhabitants of the region and of individual, mainly Spaniards, of Caucasian and/or African origin, who came to America during the sixteen century (Vargas-Alarcon et al. 2014). We excluded subjects with diabetes and patients who were undergoing treatment for dyslipidemia, hypertension, thyroid diseases or obesity with a low calorie diet to eliminate the possible effect of medications on clinical and biochemical parameters and also excluded subjects with other chronic disease and pregnant women. The study was approved by the ethics committee of the INCMNSZ, and all subjects gave written informed consent.

### Anthropometric measurements

A complete physical examination was performed on each patient. The anthropometric evaluation included measurements of the body weight, height, and waist circumference (WC) (Lohman and Martorell 1998). The percentage of fat mass and lean mass was obtained using a whole body composition analyzer (e-Body 205, Jawon Medical) in the morning after 12 h of fasting. The nutritional status of the subjects was evaluated by BMI classification according to the World Health Organization (Obesity: preventing and managing the global epidemic. Report of a WHO consultation 2000).

### Biochemical parameters

Blood samples were collected after a 12 h fasting period on the same day of elective surgery prior to surgical anesthesia. Serum total cholesterol (TC), triglycerides (TG), and HDL were determined using a Synchron CX5Δ analyzer (Beckman Coulter, CA, USA), and LDL was calculated using the Friedewald formula (Friedewald et al. 1972). Total serum adiponectin and leptin were measured using an ELISA kit (Millipore, Billerica, MA, USA). C-reactive protein (CRP) was measured by a high-sensitivity ELISA (CardioPhase hs-CRP, Siemens Health Diagnostics, Marburg Germany). Glucose was determined using the glucose oxidase method (Boehringer, Mannheim, Germany). Insulin was measured by an enzyme immunoassay (ALPCO Diagnostics, Salem NH, USA).

### Branched-chain amino acids

Serum BCAA levels were determined using a colorimetric assay kit following the manufacturer’s instructions (Abcam, Cambridge MA) with leucine as a standard.

### Genotyping

DNA was extracted from leukocytes (Miller et al. 1988). Single nucleotide polymorphisms (SNPs) of *BCKDH* rs45500792, *BCAT2* rs11548193, ATP–binding cassette transporter A1 (*ABCA1*) rs 9282541, fat mass- and obesity-associated gene (*FTO*) rs 9939609, glucose-fructose oxidoreductase domain containing 2 (*GFOD2*) rs 12449157, peroxisome proliferator activated receptor γ (*PPARγ*) rs1801282, and transcription factor 7-like 2 (*TCF7L2*) rs7903146 were determined by an allelic discrimination using a PCR endpoint TaqMan SNP Genotyping assay (ABI Prism 7900 HT Sequence Detection System, Applied Biosystems, Foster City CA, USA) (Tsai et al. 2010). These genotypes were distributed according to the Hardy–Weinberg equilibrium (Emigh 1980).

### Adipocyte area

The area of adipocytes was measured with ImageJ 1.42p digital imaging processing software (Rasband 1997–2012).

### Omental adipose tissue and adipocyte RNA isolation and real-time quantitative RT-PCR

An AT sample was obtained from a subgroup of 51 subjects, and adipocytes were isolated from AT as previously described (Frigolet et al. 2011). Total RNA was extracted from AT and adipocytes as previously described (Chomczynski and Sacchi 1987) followed by the measurement of RNA integrity, concentration and purity. The mRNA abundance was measured by real-time quantitative PCR using Taqman Assays (Applied Biosystems). Assays for each gene were conducted in triplicate in 96-well optical plates with a sequence detection system (ABI Prism 7000, Applied Biosystems). All data were normalized to housekeeping gene [low-density lipoprotein receptor-related protein 10 (*LRP10*) or cyclophilin] using the *CT* method. The relative amounts of all mRNAs for samples were calculated by the comparative *CT* method) (Livak and Schmittgen 2001). Relative mRNA expression in adipose tissue was calculated based on the efficiency (*E* = ^[−1/slope]^) of the primer/probe hybridization, and the threshold cycle (*CT*) values deviation between each sample and the control were expressed in comparison to the reference gene (*LRP10*) based on equation described previously (Tovar et al. 2011).

Patients were classified into groups according to the presence of IR or MetS. IR was evaluated and considered present when HOMA-IR was ≥2.5 (Almeda-Valdes et al. 2010). HOMA-IR was calculated as follows: HOMA = glucose (mmol/l) × insulin (pg/ml)/22.5) (Matthews et al. 1985). MetS was defined as the presence of at least three of the following factors: WC ≥ 90 cm in men and ≥80 cm in women, TG ≥ 1.7 mmol/l, HDL < 1 mmol/l in men and <1.3 mmol/l in women, systolic blood pressure ≥ 130 mmHg or diastolic blood pressure ≥ 85 mmHg, and fasting glucose ≥ 5.6 mmol/l (Alberti et al. 2009).

### Statistical analysis

Data in the text and tables are presented as the mean ± SD or median with the interquartile range in parentheses, as appropriate. Dichotomous variables were expressed as frequencies and percentages. Variables were assessed using the Kolmogorov–Smirnov test to examine the distribution type. If the data did not exhibit a normal distribution, they were logarithmically transformed prior to analysis. Student’s *t* test was used to assess the differences between the two groups, and one-factor ANOVA was used for multiple comparisons. The Pearson correlation was calculated to determine the correlation between the HOMA-IR and BMI. Significance was assumed for levels <0.05 (two-tailed). All analyses were performed using the statistical package SPSS version 12.0. The sample size required to study the gene expression of the transcription factors and proteins in adipocytes and AT was calculated for a 30 % change in the global gene expression between patients with and without IR (Tinahones et al. 2010), 80 % power and an alpha error of 0.05; a sample size of 23 patients per group was needed to meet these requirements.

## Results

### Demographic, anthropometric, and biochemical characteristics

One hundred and fifteen subjects underwent a Roux-en-Y gastric bypass for morbid obesity (51 %), Nissen fundoplication (27 %), Heller cardiomyotomy type (6 %), elective cholecystectomy (9 %), and other (7 %). Subjects had a mean age of 40 ± 10 years, and included 68 women (59 %) and 47 men (41 %). Patients were classified according to the World Health Organization BMI categories. Thus, 22 subjects had normal weight (19 %), 27 (24 %) were overweight, 8 (7 %) were class I obese, 4 (3 %) were class II obese, and 54 (47 %) were class III obese. The results of the present study were evaluated based on the presence of (1) IR, (2) MetS, (3) variants in genes related to BCAA metabolism or (4) other genetic variants.

#### Insulin resistance

**Fig. 1:**
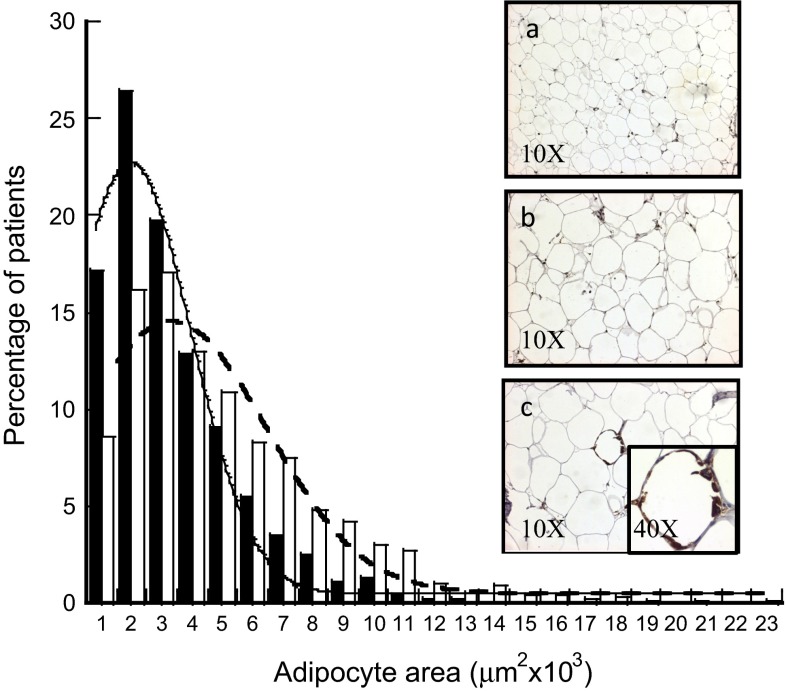
Adipocyte area from the visceral adipose tissue of insulin (IR) and non-insulin resistance (non-IR) subjects. The *white bars* represent the adipocyte area from the subjects with IR, and the *black bars* represent those with non-IR. **a** Representative photographs of the adipocyte area from lean subjects, **b** subjects with both class III obesity (BMI 40 kg/m^2^) and IR, and **c** subjects with both class III obesity (BMI 62 kg/m^2^) and IR; the photographs show increased macrophage infiltration and crown-like structures

Of 115 participants, 49 subjects were considered non-IR (42 %), and 66 subjects were classified as IR (58 %). The demographic, anthropometric, and clinical data from both non-IR and IR subjects are provided in Table 1. Patients with IR had a higher BMI, body fat mass percentage, adipocyte area, and higher levels of HOMA-IR, serum glucose, insulin, leptin, and CRP (*p* < 0.005). Interestingly, the median adipocyte area in non-IR subjects was significantly lower than that in subjects with IR (*p* < 0.005), as shown in Table 1. The Gaussian distribution curve showed that the percentage of small adipocytes was higher in non-IR subjects than in subjects with IR; conversely, subjects with IR had larger adipocytes than those of non-IR subjects, as shown in Fig. 1.

**Table 1:** Demographic, anthropometric, and serum biochemical parameters of non-insulin- and insulin-resistant subjects (*n* = 115)

Variable	Non-insulin resistant (*n* = 49)	Insulin resistant (*n* = 66)	*p* value
Gender (F/M)	27/22	41/25	NS
Age (years)	41 ± 10	39 ± 11	NS
BMI (kg/m^2^)	29.8 ± 10.9	44.2 ± 11.8	<0.001
Body fat (%)	33.6 ± 11.2	48.7 ± 6.5	<0.001
Adipocyte area (µm^2^)	3061 (1,830–4,064)	4769 (2,871–5,256)	<0.005
Glucose (mmol/l)	4.9 ± 0.6	5.8 ± 0.9	<0.001
Insulin (pmol/l)	48.8 (34.4–64.6)	166.5 (109–202)	<0.001
HOMA-IR	1.6 ± 0.6	5.4 ± 3.2	<0.001
Triglycerides (mmol/l)	2.0 (1.2–2.5)	1.9 (1.4–2.4)	NS
Cholesterol (mmol/l)	4.9 ± 1.0	4.6 ± 0.8	NS
HDL (mmol/l)	1.1 ± 0.4	1.0 ± 0.3	NS
LDL (mmol/l)	2.8 ± 0.8	2.6 ± 0.6	NS
Apo A (mmol/l)	3.5 ± 0.9	3.4 ± 0.7	NS
Apo B (mmol/l)	2.6 ± 0.7	2.5 ± 0.6	NS
Adiponectin (mg/l)	9.6 ± 5.0	8.0 ± 2.3	NS
Leptin (mg/l)	21.1 (7.1–22.5)	69.4 (26.6–102.6)	<0.001
CRP (mg/l)	40 (10–59)	57 (33–83)	<0.005

##### Gene expression of C/EBPα, PPARγ2, SREBP-1, PPARα, UCP1, leptin receptor, leptin, adiponectin, and TNFα in omental AT and adipocytes

The gene expression in adipocytes and AT was evaluated in a subgroup of 51 subjects. The demographic, biochemical, and anthropometric parameters are shown in Table 2.

**Table 2:** Demographic, anthropometrical, and serum biochemical parameters of non-insulin- and insulin-resistant subjects (*n* = 51)

Variable	Non-insulin resistant (*n* = 26)	Insulin resistant (*n* = 25)	*p* value
Gender (F/M)	13/13	14/11	NS
Age (years)	43 (38–50)	40 (32–46)	NS
BMI (kg/m^2^)	27.2 (23.9–28.8)	39.6 (30.7–53.3)	<0.001
Body fat (%)	28.0 (24.3–37.3)	50.3 (43.3–56.4)	<0.005
Adipocyte area (µm^2^)	2869 (1,933–4,361)	4,680 (2,895–6,157)	<0.05
Glucose (mmol/l)	4.8 (4.6–5.1)	5.6 (4.8–6.3)	<0.005
Insulin (pmol/l)	50.9 (36.6–66.7)	119 (108–156)	<0.001
HOMA-IR	1.7 (1.2–2.0)	4.5 (3.8–5.5)	<0.001
HDL (mmol/l)	0.90 (0.72–1.20)	0.85 (0.75–1.02)	NS
Adiponectin (mg/l)	7.3 (5.9–9.0)	5.5 (5.2–8.2)	NS
Leptin (mg/l)	16 (6.8–82.4)	75.2 (42.6–93.7)	NS
CRP (mg/l)	26 (4–56)	83 (27–100)	<0.05

**Fig. 2:**
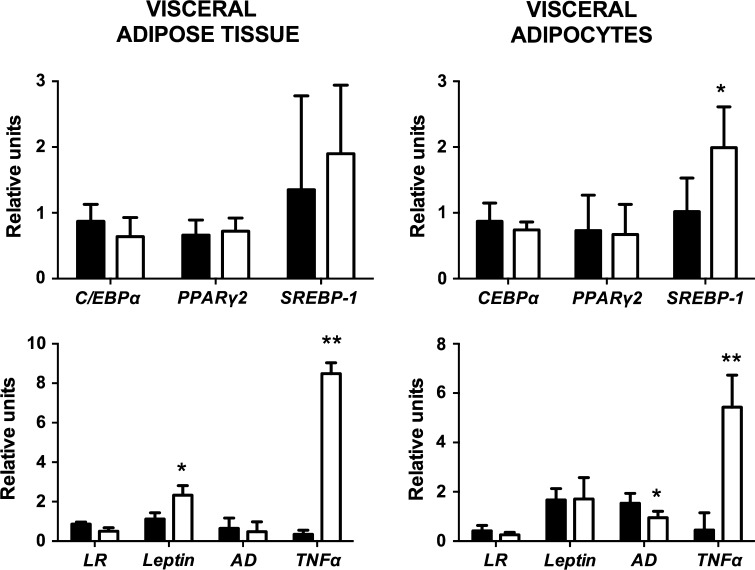
Visceral adipose tissue and adipocyte mRNA gene expression of *C/EBPα*, *PPARγ2, SREBP*-*1,* *leptin receptor* (LR), *leptin, adiponectin* (AD), and *TNFα*. *Black bars*, subjects without insulin resistance (non-IR); *white bars*, subjects with insulin resistance (IR) *p < 0.05, **p < 0.01

Figure 2 shows the mRNA levels of the different genes that were investigated in the present study. There was a large variation in the expression levels of the different target genes, and there was no difference between the different genes according to the BMI (data no shown). The expression of adipogenic and lipogenic genes was assessed in AT and adipocytes (Fig. 2a, b). No significant change was observed for C/EBPα and PPARγ2 between IR and non-IR subjects, suggesting a low level of adipogenesis in both groups. However, the expression of the *SREBP*-*1* gene in IR subjects was significantly increased in the adipocytes (Fig. 2b), indicating that increased insulin levels also increased the expression of this lipogenic transcription factor in the adipocytes. These data are consistent with the important role of *SREBP*-*1* in the expression of genes involved in fatty acid synthesis and is also consistent with the significant increase in the adipocyte area (4,680 μm^2^) compared with that in patients without IR (2,869 μm^2^).

For the genes related to fatty acid oxidation, such as *PPARα* and *UCP1,* there were no differences due to the dispersion of the values (data no shown). We compared the expression of genes involved in endocrine function and inflammation in AT and adipocytes (Fig. 2c, d). For the inflammatory and anti-inflammatory genes, there was a significantly higher expression of *TNFα* in both adipocytes and AT, lower *adiponectin* gene expression in adipocytes, and higher *leptin* gene expression in the AT of subjects with IR.

##### Serum BCAA levels and omental adipose tissue BCAT2 and BCKDH 1Eα gene expression

**Fig. 3:**
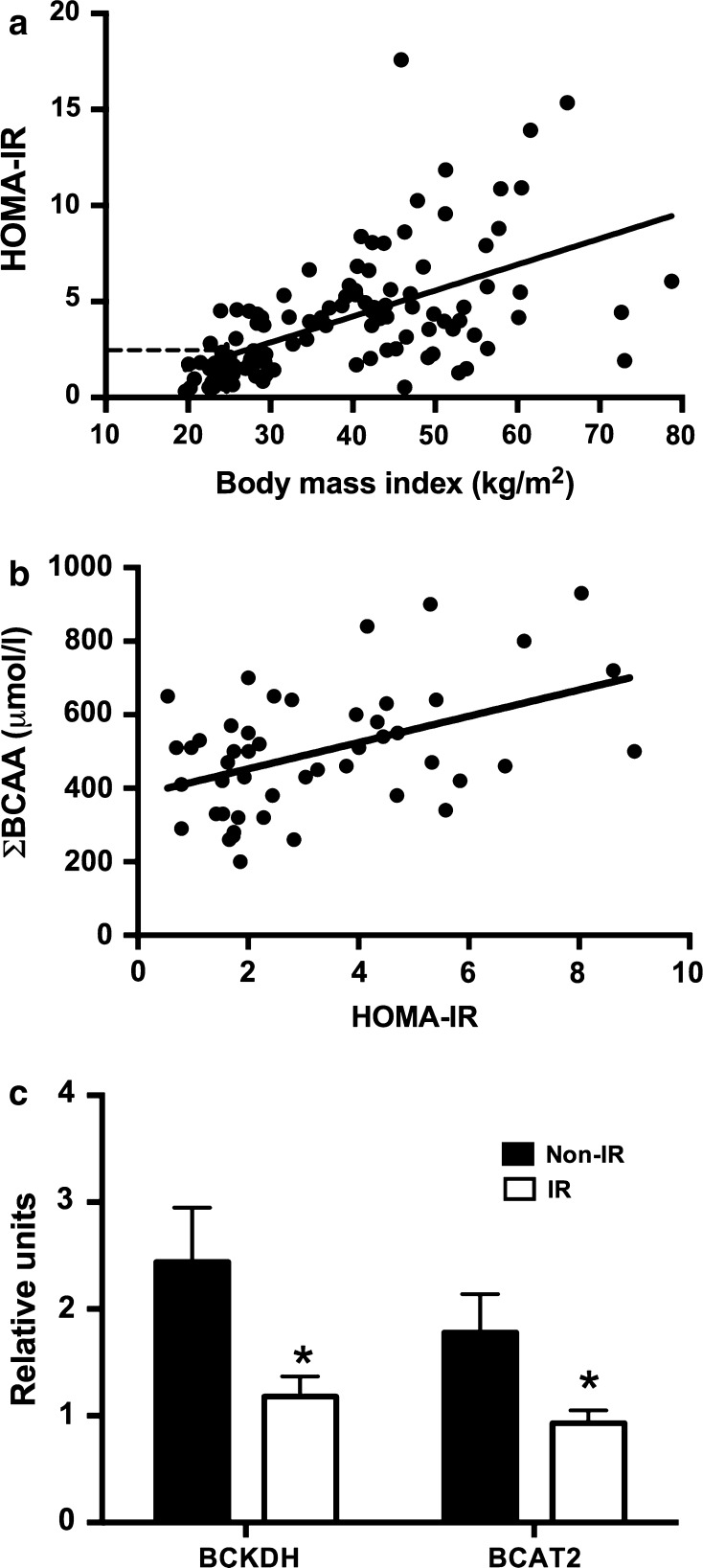
Associations between body mass index, HOMA-IR, BCCAs, omental adipose tissue BCAT, and BCKDH gene expression. **a** Bivariate correlation between HOMA-IR and BMI, *r* = 0.64 (Pearson test), *p* < 0.001. **b** Bivariate correlation between Σ BCAAs and HOMA-IR, *r* = 0.45 (Pearson test), *p* < 0.001. **c** Relationship between visceral adipose tissue gene expression of the *BCKDH* and *BCAT2* in subjects with insulin resistance (IR) and non-insulin resistance (non-IR) *p < 0.05 (**c**)

As expected, we observed a significant positive correlation between BMI and HOMA-IR (*r* = 0.64; *p* < 0.001), as shown in Fig. 3a. In addition, we found a significant positive correlation between Σ BCAA and HOMA-IR (*r* = 0.45; *p* < 0.001) as shown in Fig. 3b. Interestingly, our data clearly showed that the expression of the *BCAT2* and *BCKDH 1Eα* genes in AT was significantly reduced in subjects with IR, as shown in Fig. 3c.

#### Metabolic syndrome

**Fig. 4:**
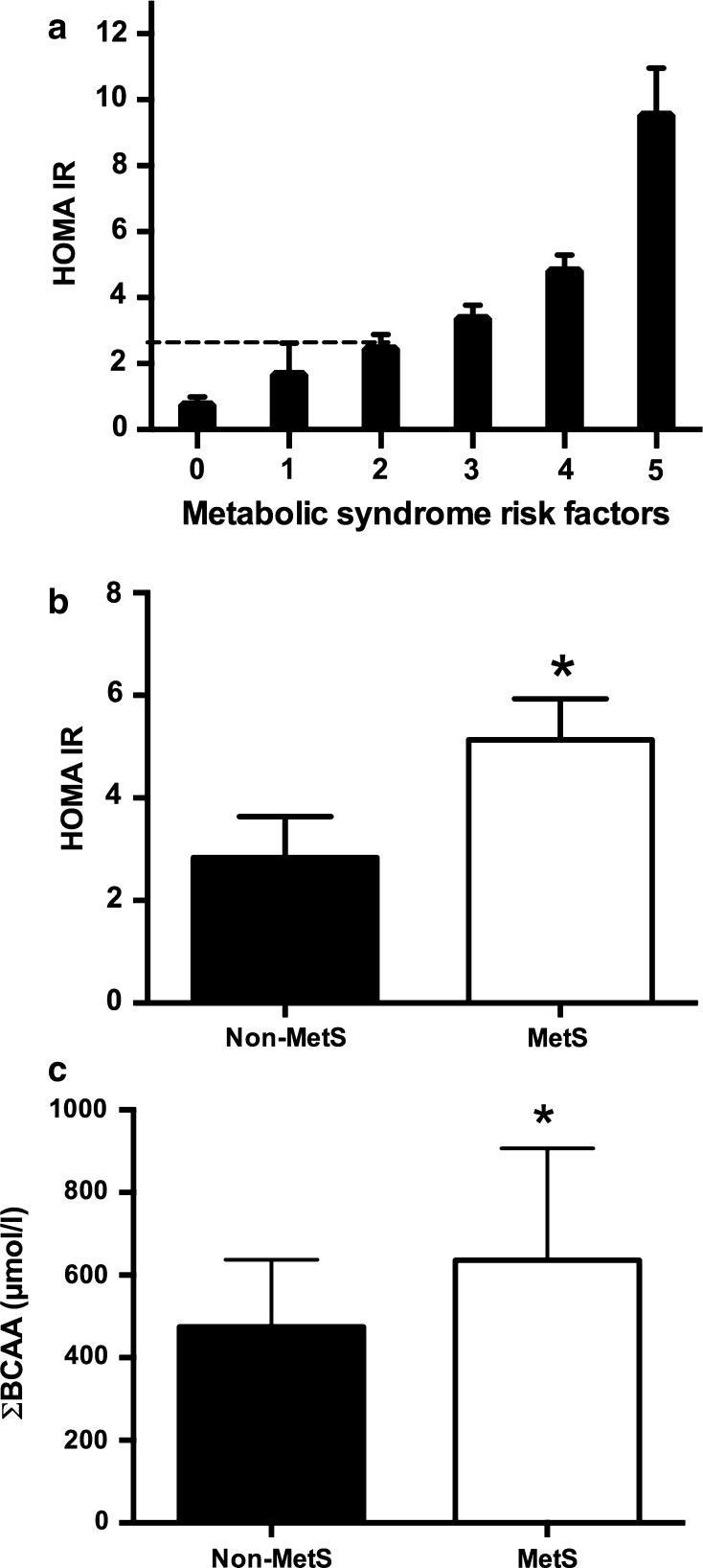
Association between HOMA-IR, metabolic syndrome and BCAA. Relationship between HOMA-IR and metabolic syndrome (MetS) risk factors (**a**). Relationship between HOMA-IR and MetS (**b**); Relationship between the Σ BCAAs and MetS (**c**). The *white bars* represent subjects with MetS, and the *black bars* represent those without MetS

Subjects were also classified according to the diagnosis of MetS. Approximately 50 % of the 115 subjects had MetS, and the most common factor in these subjects was low HDL concentration, followed by obesity, hypertriglyceridemia, hyperglycemia, and hypertension, as shown in Table 3. Interestingly, subjects with only 2 MetS factors showed IR, and those with 5 MetS risk factors had the highest HOMA-IR (9.5), Fig. 4a.

**Table 3:** Frequency of metabolic syndrome (MetS) risk factors in 115 subjects

Risk factor	Men (*n* = 47)	Women (*n* = 68)	Total (*n* = 115)
Low HDL			
Men < 1 mmol/l	35 (74 %)	47 (69 %)	82 (74 %)
Women < 1.25 mmol/l			
Waist circumference			
Men > 90 cm	30 (64 %)	50 (74 %)	80 (70 %)
Women > 80 cm			
Serum triglycerides			
≥1.65 mmol/l	33 (70 %)	23 (34 %)	56 (50 %)
Serum glucose			
≥5.5 mmol/l	16 (34 %)	25 (37 %)	41 (35 %)
Blood pressure			
>130/85 mmHg	14 (30 %)	20 (29 %)	34 (30 %)
Diagnosis of MetS	27 (57 %)	30 (44 %)	57 (50 %)

##### Serum branched-chain amino acid levels and MetS

We investigated whether subjects with MetS showed changes in the serum concentration of BCAAs. Interestingly, as shown in Fig. 4c, there was a significant increase in the serum BCAA concentration in subjects with MetS (636 ± 271 µmol/l) relative to that in the subjects without MetS (475 ± 162 µmol/l) (*p* < 0.05). This result implied that subjects with MetS had a serum BCAA concentration approximately 34 % higher than that in subjects without MetS, and as expected, subjects with MetS had significantly higher HOMA-IR, Fig. 4b.

#### BCAA metabolism gene variants

**Fig. 5:**
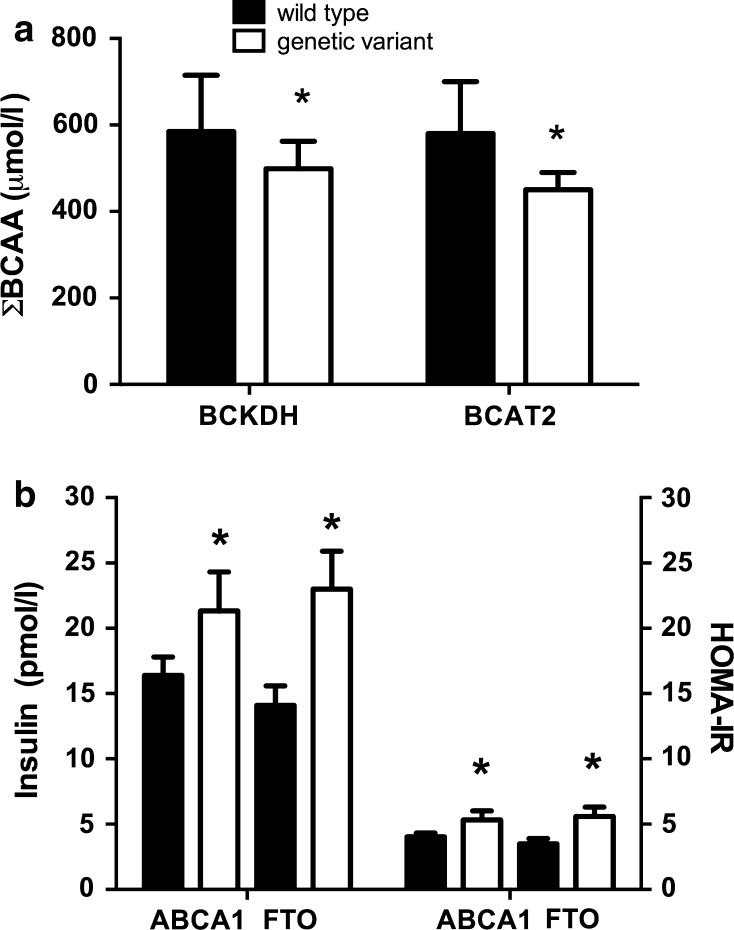
Relationship between Σ BCAAs and the presence of the genetic variants of *BCKDH* and BCAT2 *p < 0.05 (**a**). Relationship between insulin and HOMA-IR and the presence of the genetic variants of *ABCA1* and *FTO* (**b**) *p < 0.05

To understand why obese subjects with MetS who develop IR had lower AT expression of the enzymes involved in BCAA metabolism, we explored whether this finding could be partly associated with the presence of a polymorphism in *BCAT2* or *BCKDH*. We studied two SNPs of *BCAT2* and one SNP of *BCKDH*; these SNPs have a frequency higher of 10 % in HapMap (EMBL-EBI 2013). Although one *BCAT2* (rs73587806) gene variant did not show any association with the serum BCAA concentration, the subjects with the *BCAT2* (rs11548193) or *BCKDH* (rs45500792) gene variants had a significantly lower serum BCAA concentration than that of the wild-type subjects. In fact, the subjects with the *BCAT2* or the *BCKDH* gene variants showed approximately 29 or 17 %, respectively, lower serum BCAA levels compared with wild-type subjects, Fig. 5a. Interestingly, although there were no significant differences in HOMA between subjects with the gene variants for *BCAT2* or *BCKDH* compared to wild-type subjects, those with the *BCAT2* variant tended to have a lower HOMA-IR (3.08 ± 2.8) than wild-type subjects (4.35 ± 3.2).

#### Association of ABCA1, FTO, GFOD2, PPARγ, and TCF7L2 gene variants with serum insulin and HOMA-IR

To further assess whether the presence of specific SNPs with a high frequency in the Mexican population that have been associated with obesity and IR could explain the metabolic phenotype, particularly the serum insulin levels and HOMA-IR, we analyzed the SNPs for *ABCA1, FTO*, *GFOD2*, *PPARγ*, and *TCF7L2* in subjects with IR or MetS. The subjects with the presence of the *ABCA1* polymorphism and the *FTO* risk allele showed significantly higher insulin concentration and HOMA-IR compared with subjects without the variant, as shown in Fig. 5b. Interestingly, 37 % of subjects with the *FTO* risk allele were IR (*p* < 0.05), and 10 % of the subjects with the *ABCA1* polymorphism had MetS. There was no significant association between the *GFOD2, PPARγ*, *TCF7L2* polymorphisms, and IR or MetS. None of these SNPs were associated with the serum BCAA concentration. Nonetheless, only subjects with the ABCA1 polymorphism tended to have higher serum BCAA concentration (842 ± 210) than wild-type subjects (518 ± 220).

## Discussion

Obesity is a multifactorial disorder influenced by a mixture of genetic, behavioral, life-style, over nutrition, and environmental factors. These factors and their interactions cause expansion in fat mass. Our results revealed some of the factors involved in the development of the AT dysfunction associated with obesity. The overnutrition that is reflected by an increase in plasma amino acid concentrations (Tovar et al. 1996) produces an excess of amino acids that must be rapidly oxidized in the liver by the amino acid-catabolizing enzymes. However, BCAAs undergo a different metabolic fate due to the absence of *BCAT2* in the liver. BCAAs bypass the liver and are selectively metabolized in extra hepatic tissues, mainly skeletal muscle, AT and the brain (Brosnan and Brosnan 2006). Our findings and previous work (Lackey et al. 2013) indicate that omental white adipose tissue plays an important role in BCAA homeostasis because this organ has a large capacity to catabolize BCAAs, and the presence of insulin resistance or metabolic syndrome downregulates AT BCAA pathway enzyme expression. Interestingly, we observed that the higher the BMI and IR the higher the serum BCAA concentrations possibly due to a decrease in the expression of the two key BCAA enzymes, *BCAT2* and *BCKDH E1α* in AT. The results support the concept that the BCAA catabolic pathway in adipose tissue is sensitive to changes in insulin action and that insulin resistance impairs efficient BCAA catabolism in AT (Lackey et al. 2013). These results suggest that hypertrophic adipocytes develop metabolic inflexibility, which may prevent the utilization of BCAA. It has been proposed that high tissue and blood concentrations of BCAAs in human obesity cause or exacerbate IR through mechanisms involving leucine; this amino acid promotes the activation of the mechanistic target of rapamycin (*mTOR*) in muscle (Tremblay et al. 2005) and the phosphatidylinositol 3-kinase signaling pathways (Nellis et al. 2002). High serum BCAA (especially leucine) concentrations were associated with obesity and hyperinsulinemia, a finding that is consistent with earlier studies suggesting that BCAAs may augment the pancreatic secretion of insulin in the IR state (Pietilainen et al. 2008).

Because obesity is often accompanied by adipocyte metabolic dysfunction we explored other factors involved in AT dysfunction such as specific variants in the genes of the BCAT and BCKDH enzymes that could be involved in the regulation of BCAA. Little is known regarding whether the presence of gene variants of the key enzymes in BCAA catabolism is associated with serum BCAA concentration. Interestingly, our data showed that subjects with the gene variants for *BCAT2* or *BCKDH* have significantly lower serum concentrations of BCAAs. The *BCAT2* gene variant generates a BCAT2 protein containing a threonine instead of an arginine; this change can affect the substrate- or cofactor-binding sites, altering BCAT2 activity. In contrast, the change in the *BCKDH* gene variant is located in the 5′ UTR that can modify a potential response element, subsequently modifying the *BCKDH* expression. These data suggest that subjects with these gene variants may have higher enzyme activity compared with wild-type subjects; however, detailed studies are needed to understand these changes. Interestingly, we also examined several polymorphisms that included *ABCA1, FTO, PPARγ,* and *TCFL2,* but none of them were associated with serum BCAA concentration. Our results can partially explain the increase in the serum BCAA concentration of obese subjects through (1) decreased expression of the BCAA catabolism enzymes, particularly BCAT2 and BCKDH, (2) the presence of specific gene variants of *BCAT2* and *BCKDH,* and (3) the amount of fat accumulation in adipocytes that affects AT functionality.

Besides that, other specific variants can contribute to the insulin resistance. In the present study, subjects with the *ABCA1* gene variant exclusive to Native American individuals (Villarreal-Molina et al. 2007), and with the *FTO* variant showed higher values for the serum insulin concentration and HOMA-IR compared without the polymorphism. In addition, subjects with only two MetS risk factors showed IR suggesting the high susceptibility of the population studied to develop IR.

In the present study, we also observed a significant increase in the inflammatory adipokine *TNFα* and a decrease in the gene expression of *adiponectin*, which is an anti-inflammatory cytokine. IR has been associated with increased macrophage infiltration in AT and adipocyte size. However, the pathogenic link between increased AT mass and a higher risk for obesity-related disorders did not directly involve the expression of the genes associated with adipocyte differentiation, fatty acid oxidation, or thermogenesis due to individual variability. Variations in the expression of obesity-related genes in fat are influenced by the complex interactions between genes and the environment, the contributions of which are difficult to disentangle.

Enlarged adipocyte size is associated with IR and increased adipokine production and secretion. In our study, IR was associated with increased adipocyte area and increased AT mass; these changes occurred due to inadequate vascularization and thus might lead to hypoxia, macrophage infiltration, and inflammation. The adipokines, secreted by hypertrophied adipocytes, attract macrophages that are dispersed throughout the tissue or clustered around adipocytes in “crown like structures”; these macrophages secrete cytokines that contribute to the low-grade inflammation seen in obesity. Chronic inflammation is another hallmark of unhealthy adipocytes and is most likely a major mechanism responsible for lowering HDL during obesity (McGillicuddy et al. 2011).

**Fig. 6:**
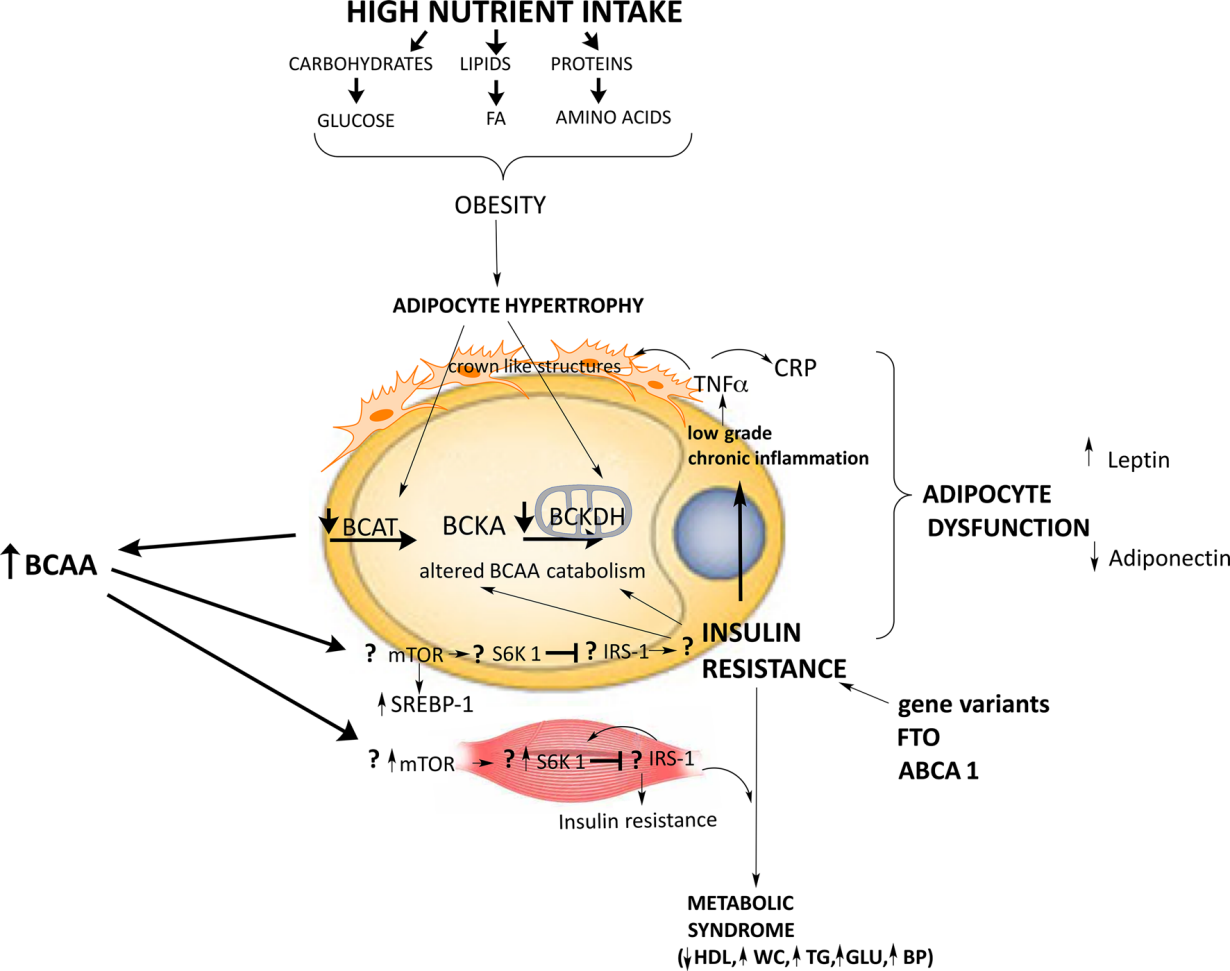
Summary of the factors (e.g., genetic factors, biochemical factors, endocrine factors, and inflammatory markers) and physiological differences, gene expression, and environmental interactions cause AT dysfunction by initiating a sequence leading to adipocyte hypertrophy and inflammatory processes

AT dysfunction is a primary defect in human obesity, which involves complex pathophysiological mechanisms that evolve over time and are largely beyond individual control, and may link obesity to several health problems, including increased risk for type 2 diabetes, fatty liver, and cardiovascular disease. Here, we propose a process for how different factors (e.g., genetic factors, BCAA metabolism, endocrine factors, and inflammatory markers) and physiological differences, gene expression, and environmental interactions cause AT dysfunction by initiating a sequence leading to adipocyte hypertrophy and inflammatory processes, Fig. 6. As a consequence, impaired AT function contributes to a proinflammatory, atherogenic, and diabetogenic state and may be mechanistically linked to the development of obesity-associated disorders. The complexity of obesity, along with the potential for disease progression and the heterogeneity of individuals with obesity, must be considered when designing treatment strategies.
